# Recent Configurational Advances for Solid-State Lithium Batteries Featuring Conversion-Type Cathodes

**DOI:** 10.3390/molecules28124579

**Published:** 2023-06-06

**Authors:** Kuan-Cheng Chiu, Jeng-Kuei Chang, Yu-Sheng Su

**Affiliations:** 1 International College of Semiconductor Technology, National Yang Ming Chiao Tung University, 1001 University Road, Hsinchu 30010, Taiwan; 2 Department of Materials Science and Engineering, National Yang Ming Chiao Tung University, 1001 University Road, Hsinchu 30010, Taiwan; 3 Industry Academia Innovation School, National Yang Ming Chiao Tung University, 1001 University Road, Hsinchu 30010, Taiwan

**Keywords:** all-solid-state battery, metallic lithium anode, chalcogen cathode, chalcogenide cathode, halide cathode, solid-state electrolyte, Li–S battery, sulfur cathode, sulfide cathode, fluoride cathode

## Abstract

Solid-state lithium metal batteries offer superior energy density, longer lifespan, and enhanced safety compared to traditional liquid-electrolyte batteries. Their development has the potential to revolutionize battery technology, including the creation of electric vehicles with extended ranges and smaller more efficient portable devices. The employment of metallic lithium as the negative electrode allows the use of Li-free positive electrode materials, expanding the range of cathode choices and increasing the diversity of solid-state battery design options. In this review, we present recent developments in the configuration of solid-state lithium batteries with conversion-type cathodes, which cannot be paired with conventional graphite or advanced silicon anodes due to the lack of active lithium. Recent advancements in electrode and cell configuration have resulted in significant improvements in solid-state batteries with chalcogen, chalcogenide, and halide cathodes, including improved energy density, better rate capability, longer cycle life, and other notable benefits. To fully leverage the benefits of lithium metal anodes in solid-state batteries, high-capacity conversion-type cathodes are necessary. While challenges remain in optimizing the interface between solid-state electrolytes and conversion-type cathodes, this area of research presents significant opportunities for the development of improved battery systems and will require continued efforts to overcome these challenges.

## 1. Introduction

Rechargeable lithium-ion batteries have dominated major energy storage battery applications for the past decade, including electric vehicles, drones, consumer electronics, and stationary and mobile energy storage systems. Traditional lithium-ion batteries consist of graphitic anodes, polyolefin separators, organic liquid electrolytes, and intercalation-type lithium transition metal oxides/phosphate cathodes. Among these, the cathode material is the key component that limits the energy density of lithium-ion/lithium metal batteries [1,2,3]. Therefore, conversion-type cathode materials are in the spotlight of battery material researchers because of their high gravimetric and volumetric capacity for lithium-ion storage [4,5].

**Figure 1:**
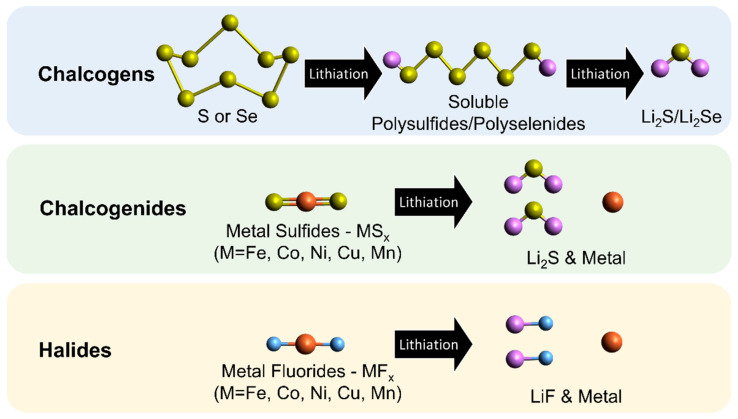
Schematic drawing of the lithiation reaction of conversion-type cathodes.

The high-energy-density conversion-type cathode materials for lithium batteries can be divided into three main categories: chalcogens, chalcogenides, and halides. Figure 1 displays how lithium ions react with these cathodes during the conversion-type lithiation, and they can be reversibly transformed back to their initial states under a rational electrode design. When these conversion-type cathodes are cycled in the cell with a liquid electrolyte, dissolution of active materials can occur, inducing undesirable shuttle reactions that result in poor cycle life [5,6,7]. Chalcogen cathode materials form soluble intermediates (high-order polysulfides or polyselenides) at the early stage of lithiation and then convert into lithium sulfide/selenide as the final product upon further discharge [8,9,10]. The most investigated chalcogen cathode material is sulfur, which has been optimized using various state-of-the-art electrodes and cell architectures in Li–S cells with a liquid electrolyte [6,11]. Selenium has a much higher electrical conductivity than sulfur, leading to a potentially better power performance [12], but its high material cost and low earth abundance may hinder its commercial viability [5].

The second category of conversion-type cathodes are chalcogenides, mostly coupled with transition metals (Fe, Co, Ni, Cu, Mn) [5,13]. Taking metal sulfides as an example, the cathode compound is converted to metal and lithium sulfide (Li_2_S) after lithiation and vice versa after delithiation [14,15,16,17]. The chalcogenide anions can be replaced by halide anions as the third type of conversion cathode. Fluoride cathode materials are popular in this category, forming intermediate lithiated metal fluoride compounds (Li_x_M_y_F_z_), metal, and lithium fluoride (LiF) instead of Li_2_S after the discharge reactions [18,19,20,21]. In fact, FeF_3_ has multistep and mixed intercalation/conversion reactions during lithiation and delithiation [18,19], which is still included in this review. All three types of conversion cathode materials must be paired with lithiated anodes, such as metallic lithium, prelithiated graphite, and prelithiated silicon, to make a full cell, making them relatively difficult to process compared to traditional lithium-ion batteries. Oxygen cathodes have received considerable attention due to their environmentally friendly nature. This has led to a particular interest in their conversion reaction chemistry, especially when paired with solid-state electrolytes [22,23,24,25]. Compared to other solid-state conversion cathodes, oxygen cathodes have fundamentally different technological barriers due to the gas–solid conversion reactions and catalyst requirements. However, since several review articles have already covered Li–O_2_ batteries, we will not delve into the specifics of this chemistry but will focus only on chacolgen, chacolgenide, and halide cathodes in this review.

**Figure 2:**
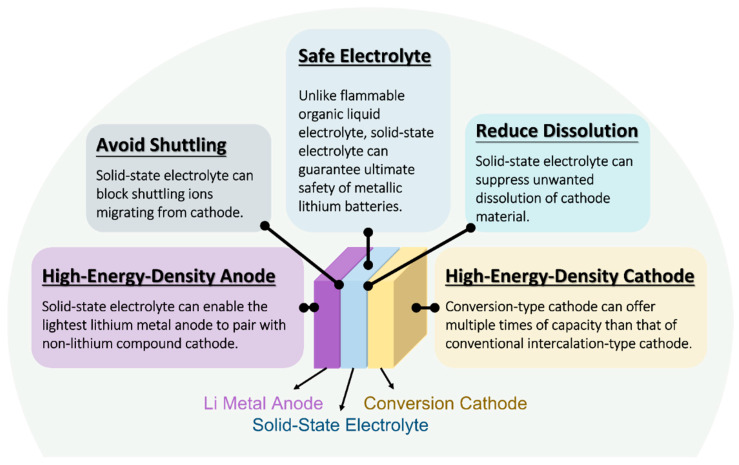
Schematic illustration of the advantages of using conversion-type cathodes in lithium solid-state batteries.

Figure 2 illustrates the advantages of integrating conversion-type cathodes with solid-state electrolytes in lithium batteries. The solid-state electrolyte can enable high-energy-density metallic lithium anodes, which are considered to have extremely poor cyclability in liquid electrolytes [8,26,27,28]. Lithium metal anodes are necessary for the non-lithiated conversion cathodes to be operated in a battery. By combining both high-energy-density electrodes, the battery can offer ~two times higher specific energy calculated based at the cell level [5]. By utilizing solid-state electrolytes instead of flammable organic liquid electrolyte, the fire hazards of the battery resulting from the low flash point of liquid electrolytes and the failure of low-melting-point polyolefin separators can be eliminated [29,30,31,32,33,34]. In addition, the absence of solvents in the cathode region can significantly solve the active material dissolution problem [35,36,37]. The notorious shuttle effect in Li–S batteries, caused by the migration of soluble polysulfides, can also be excluded in a solid-state battery [38,39]. However, the dissolution of polysulfide intermediates is required to achieve high power density and high active material utilization in Li–S batteries [40,41], which will be discussed later in the case of the solid-state configuration.

## 2. Challenges Remained in Conversion-Type Cathode Materials

Although conversion-type cathode materials have tremendous potential in terms of high gravimetric capacity, safe operating voltage, scalable synthesis routes, etc., there are still several obstacles to the commercialization of these cathode systems. In Li–S cells, poor electrical and ionic conductivities of sulfur and significant cathode volume change (from sulfur to lithium sulfide) can be addressed by intelligent 3D structural design, which is mainly enabled by carbonaceous materials and other functional materials that can accommodate and trap sulfur species [6,11,42]. Nevertheless, despite efforts to mitigate the rapid capacity degradation caused by the loss of soluble polysulfide intermediates, this problem cannot be completely solved. Moreover, there are currently no commercially viable solutions to the poor reversibility of the lithium metal anode in liquid electrolytes. In this regard, the integration of solid-state electrolyte into chalcogen cathode lithium batteries seems to be a rational strategy to mitigate the dissolution of active species in the traditional lithium battery with a liquid electrolyte [39,43,44].

For metal sulfide and metal fluoride cathode materials, their common processing problem is that they are both sensitive to moisture [45,46,47], which shares the same challenge with many solid-state electrolyte systems [48,49,50,51,52]. High electrical resistance and sluggish reaction kinetics of sulfide and fluoride materials are the main causes of their large voltage hysteresis in lithium batteries [4,5,7,18,53]. In addition, a non-uniform cathode–electrolyte interface (CEI) may be generated by the catalytic reaction during cycling, which is not strong enough to withstand the volume expansion of conversion-type cathodes [53,54]. The use of solid-state battery design can reduce the formation of unstable solid–electrolyte interface (SEI) at either the anode or the cathode (i.e., CEI) made from decomposed electrolyte components, which can improve the cycling stability of the battery with a chalcogenide or halide cathode. Other challenges, such as high overpotentials, gas generation from decomposed electrolyte ingredients, and safety concerns, also need to be addressed before the conversion-type cathodes can be implemented in commercial batteries [55,56,57].

## 3. Solid-State Lithium Battery with Conversion-Type Cathodes

### 3.1. Chalcogen Cathodes with Solid-State Electrolytes

**Figure 3:**
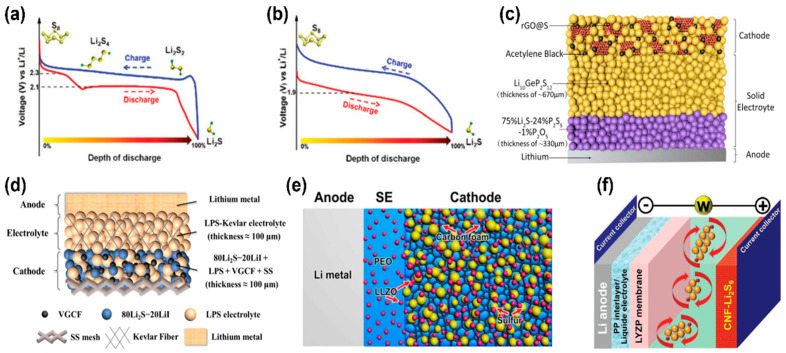
The charge/discharge voltage curves of a Li–S battery with (**a**) an ether-based liquid electrolyte and (**b**) a carbonate-based liquid electrolyte or a solid-state electrolyte. Reproduced with permission from ref. [61]. Copyright 2020, Royal Society of Chemistry (London, UK). (**c**) Schematic drawing of an all-solid-state lithium-sulfur battery with a well-mixed sulfur cathode. Reproduced with permission from ref. [66]. Copyright 2017, Wiley-VCH (Weinheim, Germany). (**d**) Schematic drawing of a cathode-supported and Kevlar fiber-reinforced all-solid-state Li−Li_2_S cell. Reproduced with permission from ref. [67]. Copyright 2019, ACS Publications (Washington, DC, USA). (**e**) Schematic drawing of an all-solid-state Li–S battery with a hybrid LLZO/PEO electrolyte system. Reproduced with permission from ref. [68]. Copyright 2017, ACS Publications (Washington, DC, USA). (**f**) Schematic drawing of a hybrid Li||LYZP||Li_2_S_6_ cell with the liquid electrolyte on both sides of the LYZP membrane. Reproduced with permission from ref. [78]. Copyright 2016, Wiley-VCH (Weinheim, Germany).

The most popular chalcogen cathode material, sulfur, is attractive due to its high theoretical energy density, low cost, and environmental friendliness [6,11]. However, Li–S batteries with a liquid electrolyte have been limited by several challenges, such as short cycle life and poor rate performance [58,59,60]. Figure 3a exhibits the typical charge/discharge profiles of a Li–S cell adopting a liquid electrolyte. Two distinct discharge plateaus represent the polysulfide dissolution reactions (upper plateau; S_8_ → Li_2_S_4_; ~2.3 V) and solid-state reactions (lower plateau; Li_2_S_4_ → Li_2_S; ~2.1 V) [10,40,61,62]. In contrast, the solid-state Li–S battery shows only a sloping curve for the reaction of sulfur converting to lithium sulfide (Figure 3b). The same behavior can also be found in the liquid-phase carbonate electrolytes because carbonates cannot dissolve polysulfide intermediates [63,64,65]. The slow kinetics resulting from the solid-state reaction mechanism occurring in Li–S solid-state batteries may hinder their practicality due to sluggish diffusion and limited interfacial contact area, which can severely degrade their performance.

To reduce the charge-transfer resistance, similar to the strategy adopted in other solid-state batteries, sulfur or lithium sulfide active materials must be blended with solid-state electrolyte particles in the cathode to achieve better utilization [66,67,68,69]. Xu et al. demonstrated a well-mixed cathode consisting of reduced graphene oxide coated with sulfur (rGO@S)/acetylene black (AB)/Li_10_GeP_2_S_12_ (LGPS) via long-duration ball-milling (Figure 3c), delivering an impressive reversible capacity of 830 mA h g^−1^ after 750 cycles at a rate of 1 C and 60 °C [66]. The incorporation of rGO@S nanocomposite into the LGPS-AB matrix results in a homogeneous distribution of the composite cathode, which facilitates uniform volume changes during lithiation/delithiation. The high cathode uniformity significantly reduces stress and strain within the solid-state cells, thereby prolonging their cycle life. Additionally, to address the issue of bulky solid-state electrolyte, Wang et al. developed a cathode-supported solid-state electrolyte configuration shown in Figure 3d, which not only reduces the ion diffusion distance between the anode and cathode but also significantly enhances the energy density of the solid-state Li–S battery (370.6 W h kg^−1^) [67]. The cathode/electrolyte/anode laminated structure was accomplished by using a stainless steel mesh-supported Li_2_S cathode as a starting point, followed by adding a robust Kevlar nonwoven scaffold-reinforced Li_3_PS_4_ (LPS) electrolyte as the top layer, with a thickness of approximately 100 μm and a metallic lithium anode [67].

One alternative approach to addressing the interfacial challenges between the cathode and solid-state electrolyte is to adopt hybrid electrolyte systems. Cui et al. utilized Li_7_La_3_Zr_2_O_12_ (LLZO) nanoparticles-filled poly(ethylene oxide) (PEO) polymer electrolyte in solid-state Li–S batteries (Figure 3e) to achieve an outstanding specific capacity of >900 mA h g^–1^ at human body temperature of 37 °C [68]. The remarkable electrochemical performance is attributed to the composite cathode and solid-state electrolyte where the LLZO nanoparticle serves both as a filler to enhance ion conductivity and as an interfacial stabilizer to mitigate interfacial resistance. Efficient ion transport in solid-state batteries depends on low interfacial resistance, which can facilitate electrochemical reactions and reduce the barrier for ions to cross the heterogeneous solid-state electrolyte/electrode interface [70,71,72]. On the other hand, PEO offers reasonable mechanical stability, good electrode compatibility, and excellent film-forming properties for the composite solid-state electrolyte [73,74]. Polymer electrolytes can fill in the interparticle voids generated at the cathode and solid-state electrolyte regions, effectively promoting interfacial wetting and enabling stable cycling of lithium electrodeposition and electrostripping at relatively low overpotentials [75,76,77]. Figure 3f shows another Li–S battery configuration adopting a hybrid electrolyte system, including a sodium (Na) super ionic conductor (NaSICON)-type solid electrolyte (Li_1+x_Y_x_Zr_2−x_(PO_4_)_3_ (LYZP) (x = 0–0.15)) and a liquid electrolyte [78,79]. By integrating a solid electrolyte with a liquid electrolyte, the hybrid dual-electrolyte Li–S battery exhibits significantly improved cyclability compared to conventional Li–S batteries that utilize a porous polymer separator immersed with a liquid-phase electrolyte [78,80]. Here, polysulfides can still dissolve in the liquid electrolyte like a catholyte and perform two-step electrochemical reactions to guarantee high active material utilization and reasonable rate performance. In summary, the solid electrolyte membrane demonstrates promising characteristics such as reasonable Li^+^ ion conductivity, superior polysulfide retention, chemical compatibility with cell components, and electrochemical stability under repeated charge-discharge conditions in Li–S cells [78,80].

**Figure 4:**
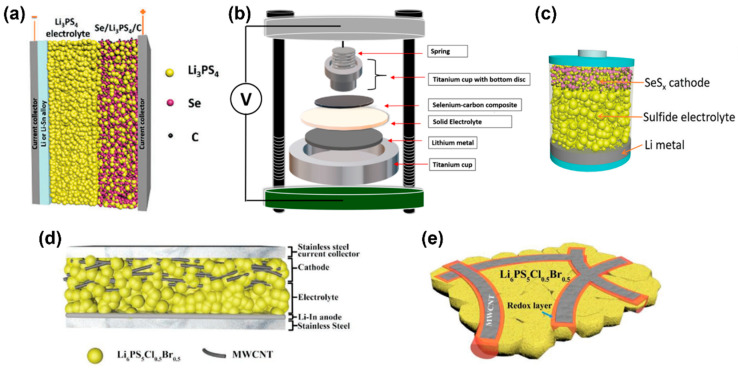
(**a**) Schematic drawing of an all-solid-state Li–Se battery. Reproduced with permission from ref. [87]. Copyright 2018, Royal Society of Chemistry (London, UK). (**b**) Cell configuration of the molten Li–Se cell tested at 465 °C. Reproduced with permission from ref. [88]. Copyright 2021, ACS Publications (Washington, DC, USA). (**c**) Schematic drawing of a Li–SeS_x_ solid-state battery. Reproduced with permission from ref. [61]. Copyright 2020, Royal Society of Chemistry (London, UK). (**d**) Schematic drawing of an Li-In||LPSCB||LPSCB-MWCNTs cell with a monolithic structure. (**e**) Schematic drawing of the LPSCB-MWCNTs composite cathode. Reproduced with permission from ref. [90]. Copyright 2021, Wiley-VCH (Weinheim, Germany).

As a cathode active material, the insulating nature of sulfur is always problematic during electrochemical reactions, especially in solid-state mechanisms. Much effort has been expended in the past to improve the contact between sulfur species and carbon substrates for better cyclability [81,82,83,84,85]. To improve the contact between the conductive carbon substrate and the sulfur, a simple heating process can be used. This involves coating the carbon surface uniformly with elemental sulfur, which promotes surface-to-surface contact. As the sulfur melts during the heating process and then solidifies after cooling, it forms a thin, uniform layer that adheres to the carbon surface. This layer facilitates the transfer of electrons between the carbon and sulfur, leading to more efficient active material utilization [86]. Substituting selenium for sulfur is a common method used to improve the intrinsic electrical conductivity of chalcogen cathode materials. Figure 4a shows an all-solid-state Li–Se battery configuration with LPS as the electrolyte [87]. The use of selenium in the cathode provides high electrical conductivity (1 × 10^−3^ S cm^−1^), while a high Li^+^ conductivity of 1.4 × 10^−5^ S cm^−1^ is achieved across the Se-LPS interface. This battery has a high reversible capacity of 652 mA h g^−1^ (96% of theoretical capacity) and exhibits favorable capacity retention during cycling. Kumar et al. also showcased the functionality of a high-temperature molten Li–Se battery cell with a garnet-type solid-state electrolyte (LLZO) operating at 465 °C (Figure 4b), which is essential for powering future space exploration missions [88]. The cells demonstrated a stable open-circuit voltage for 17 h and were subjected to electrochemical cycling at various current rates. Since selenium is a relatively high-cost material, introducing selenium into sulfur cathodes through the formation of SeS_x_ solid solutions could be an alternative means to modify the electrical and ionic conductivities of the cathode without a significant increase in material price [89]. Figure 4c illustrates the solid-state cell configuration with SeS_x_ cathode, sulfide-based solid electrolyte (LPS or LGPS), and Li metal. The use of SeS_2_ in high loading cells has been found to achieve an ultrahigh areal capacity of up to 12.6 mA h cm^−2^ [61,89]. The SeS_x_ solid solution cathode confirms the importance of cathode ionic and electrical conductivities in determining electrochemical performance.

In addition to the high ionic conductivity of sulfide-based solid-state electrolyte, lithium argyrodite sulfide (Li_6_PS_5_Cl_0.5_Br_0.5_, LPSCB) offers the advantage of serving a bifunctional role as both a solid electrolyte and a precursor material for the chalcogen cathode [90,91]. In Figure 4d, a monolithic cell configuration that uses the LPSCB material all over the electrolyte and cathode region was designed, and the LPSCB in the cathode region, along with multiwall carbon nanotubes (MWCNTs), forms a multiphase conversion-type cathode by partial decomposition during the first discharge. Meanwhile, the remaining LPSCB electrolyte remains unchanged and provides low-impedance ionic transport pathways, which enhances the cathode performance. The discharge capacity of the all-solid-state cell exhibits an initial sharp incline, followed by a gradual increase as the MWCNT content is increased [90]. This observation suggests that the active material formation occurs exclusively at the interface between the MWCNTs and the electrolyte particles, as illustrated in Figure 4e [90,92]. This finding paves the way for the development of high-performance all-solid-state batteries using thiophosphate solid electrolytes where the high cycling stability can be attributed to the intimate contact between the electrochemically reduced cathode and electrolyte interface.

### 3.2. Chalcogenide Cathodes with Solid-State Electrolytes

**Figure 5:**
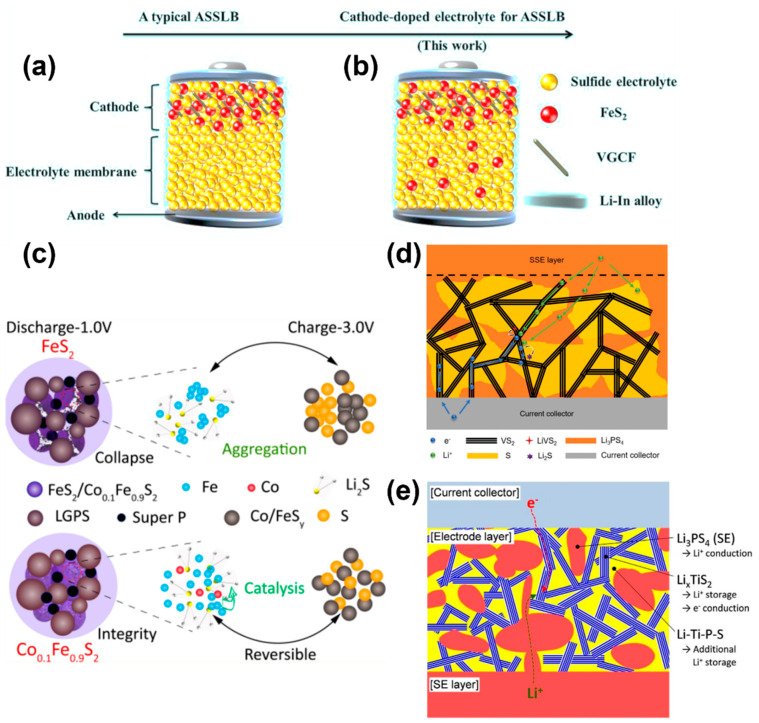
Schematic drawing of an all-solid-state lithium battery (**a**) without cathode-doped electrolyte and (**b**) with cathode-doped electrolyte. Reproduced with permission from ref. [100]. Copyright 2020, Elsevier (Amsterdam, The Netherlands). (**c**) Schematic drawing of the redox reactions of FeS_2_ and Co_0.1_Fe_0.9_S_2_ cathodes. Reproduced with permission from ref. [101]. Copyright 2019, ACS Publications (Washington, DC, USA). (**d**) Schematic drawing of the solid-state hybrid Li–S/VS_2_/LPS battery. Reproduced with permission from ref. [108]. Copyright 2021, Wiley-VCH (Weinheim, Germany). (**e**) Schematic drawing of the solid-state hybrid Li–TiS_2_/LPS battery. Reproduced with permission from ref. [109]. Copyright 2014, Springer (Berlin/Heidelberg, Germany).

Pyrite (FeS_2_) shows promise as an electrode material for lithium-ion batteries due to its natural abundance, low cost (commercialized by Energizer, St. Louis, MO, USA), non-toxicity, and ultrahigh theoretical energy density of 1313 W h kg^−1^ [93,94,95]. Moreover, recent studies have reported improved electrochemical properties in all-solid-state secondary Li/FeS_2_ batteries [96,97,98,99]. Yang et al. investigated the usage of FeS_2_ as a dopant for Li_7_P_3_S_11_-type glass-ceramic electrolytes, which has been shown to enhance ionic conductivity while reducing interfacial resistance between the FeS_2_ cathode and electrolyte (Figure 5a,b) [100]. This design was different from previous reports where a conventional FeS_2_ cathode was doped into the sulfide electrolyte by a suitable proportion (99.5(70Li_2_S–30P_2_S_5_)–0.5FeS_2_) and the largest crystallinity was obtained, boosting the ionic conductivity of the electrolyte [100]. The FeS_2_ composite cathode and sulfide electrolyte had similar chemical potential, resulting in lower interfacial resistance and superior cycling stability. Although all-solid-state batteries have the capability to support reversible four-lithium-ion storage for FeS_2_ (FeS_2_ + 4 Li = Fe + 2 Li_2_S), issues such as strain/stress concentration resulting in electrode pulverization and sluggish electrochemical reactions between lithium sulfide and sulfur can impact the long-term cycling stability of the battery [101]. Figure 5c represents an approach to utilize the loose-structured Co_0.1_Fe_0.9_S_2_-based all-solid-state lithium batteries that have been optimized via nanoengineering to achieve impressive electrochemical performance. After initial charging to 3.0 V, it was observed that the element cobalt had an extremely homogeneous distribution with iron and sulfur, and no Fe/S aggregation was detected [101]. This indicates that cobalt has a catalytic effect on the electrochemical reaction, which improves the reaction between Li_2_S and Fe. Moreover, previous studies have reported that transition metals and their metal sulfides can effectively enhance the redox reaction kinetics and reduce the shuttle effect in lithium−sulfur batteries. This is attributed to the catalytic properties of these materials and their strong ability to absorb polysulfides [102,103].

Certain chalcogenide materials, including vanadium sulfide (VS_2_) and titanium sulfide (TiS_2_), exhibit exceptional electrical conductivity, making them suitable for use as efficient electrode materials [104,105,106,107]. Figure 5d demonstrates a high-performance solid-state battery with a VS_2_/S nanocomposite cathode, which combines intercalation-type vanadium sulfide with conversion-type sulfur chemistry [108]. A facile, low-cost, and low-energy mechanical blending process was implemented to prepare the S/VS_2_/LPS composite cathode [108]. The VS_2_ nanomaterial has a layered structure with fast Li-ion transport channels, metallic conductivity, and extra capacity contribution, providing an ideal platform for the solid-state S/Li_2_S redox couple to achieve its high gravimetric capacity. A similar idea was adopted by Jung et al., demonstrating that controlled ball-milling of a sulfide-based active material (TiS_2_) and a solid-state electrolyte (LPS) for the electrode led to a significant increase in capacity in all-solid-state lithium batteries without compromising the ionic and electronic conduction pathways [109]. The increased Li^+^ ion storage is believed to be associated with the formation of an amorphous Li–Ti–P–S phase during the controlled ball-milling process. The aforementioned structural designs indicate that utilizing a mixed ion/electron conductive transition metal sulfide and an active cathode material in an all-solid-state cell configuration is a promising strategy for the development of next-generation solid-state batteries.

### 3.3. Halide Cathodes with Solid-State Electrolytes

**Figure 6:**
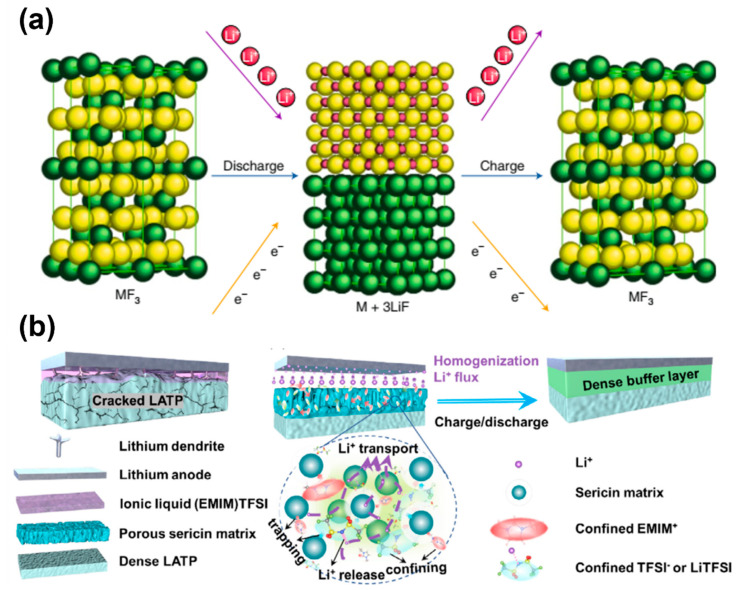
(**a**) Schematic drawing of the crystallographic reaction mechanism that occurs during the discharge of metal fluoride electrodes. Reproduced with permission from ref. [116]. Copyright 2007, Elsevier (Amsterdam, The Netherlands). (**b**) Schematic drawing of interface environment of Li/IL-LATP (**left**) and Li/IL@SP-LATP (**right**) during cycling process. Reproduced with permission from ref. [128]. Copyright 2022, Elsevier (Amsterdam, The Netherlands).

Although intercalation-type oxide cathodes and conversion-type chalcogen/chalcogenide cathodes have high capacity, their voltage output is relatively low, limiting their potential to achieve high energy density as positive electrodes. However, early studies have shown that metal fluorides can be used in the conversion process, enabling higher voltage materials through the use of nanomaterials and composites (as shown in Figure 6a) [110,111,112]. The voltage of these materials is about 1 V higher than that of chalcogens/chalcogenides. Metal fluorides thus offer a promising means towards achieving both specific and volumetric energy densities that greatly surpass the theoretical limits of current positive electrode materials (lithium cobalt oxides, lithium nickel manganese cobalt oxides, lithium nickel cobalt aluminum oxides, and lithium iron phosphates) [113,114,115]. Halides have an intrinsic advantage of exhibiting extraordinarily specific and volumetric energy density while operating at moderate voltages compared to those of commercial intercalation-type cathodes. This allows for the use of various solid and liquid electrolytes that are not stable at the higher voltages (over 4 V) used with traditional positive electrodes. In addition, previous reports suggested that the lack of active oxygen in halides leads to improved safety compared to conventional layered oxides [116].

Halide cathodes, like other conversion-type cathode materials, face agglomeration challenges that can lead to increased electrical/ionic resistance, ultimately deteriorating battery performance [117,118,119]. Numerous efforts have been dedicated to developing carbon/metal fluoride nanocomposites in order to mitigate active material aggregation [120,121,122,123,124]. Another promising approach to prevent agglomeration is through the use of surfactants [125,126]. The aforementioned drawback of increased impedance in halide cathodes can be exacerbated when they are combined with a solid-state electrolyte, as this introduces additional solid–solid interfacial resistance and potential solid-state reactions. When two materials come into contact, the difference in their standard chemical potentials drives the flow of free ions across their interface, which could potentially lead to redox reactions [127]. To the best of our knowledge, no prior research has been conducted on the interfacial reactions between the solid-state electrolyte and halide cathode surface up until now. In the case of the interface between Li_1.3_Al_0.3_Ti_1.7_(PO_4_)_3_ (LATP) and Li metal, this flow is influenced by the fact that the Li chemical potential of LATP (−4.3 eV) is lower than that of Li metal (0 eV). As a result, Li^+^ ions may transfer from Li metal to LATP upon contact, while electrons are injected from Li metal into the Ti 3d unoccupied orbital in LATP, resulting in the reduction of Ti^4+^ to Ti^3+^, followed by structural cracks (Figure 6b) [128,129]. Li et al. developed a sericin protein (SP) buffer layer with the confined ionic liquid, which can assist in minimizing the contact between ionic liquid (IL) electrolyte and Li metal, improve the solidification of ionic liquid, and ensure a homogenous dispersion of Li-ion flux at the anode/solid-state electrolyte interface. Consequently, it promotes smooth Li deposition and mitigates side reactions that may occur. Additionally, the intermolecular interaction between TFSI^-^ anions and sericin-chain can also alleviate the reduction of free TFSI^−^ by Li metal, which prevents the fast accumulation of SEI and evident passivation of symmetric cells [128]. The use of the sericin protein film protected LATP solid-state electrolyte has been shown to facilitate the successful reversible operation of Li/FeF_3_ conversion solid-state batteries.

Table 1 summarizes the new cell configurations discussed in this review. Operating temperatures play a critical role in the practical applications of solid-state batteries. Most of the reported battery systems can be operated at room temperature or near body temperature. However, there are a few exceptions. The amorphous sulfur-coated reduced graphene cathode paired with a sulfide-based solid-state electrolyte requires heating to 60 °C. Despite the elevated temperature, this cell shows excellent performance in terms of capacity and cycle life at a high rate of 1 C [66]. On the other hand, a Li–Se battery designed for space applications must be operated at a much higher temperature of 465 °C [88]. As for chalcogenide and halide cathode systems, their cycle life in solid-state batteries is still under development, and their rate performance is not yet satisfactory. In conclusion, while most solid-state batteries can operate effectively at moderate temperatures, certain configurations require higher operating temperatures. However, chalcogenide and halide cathode systems require further development to improve their cycle life and rate performance.

## 4. Summary and Outlook

**Figure 7:**
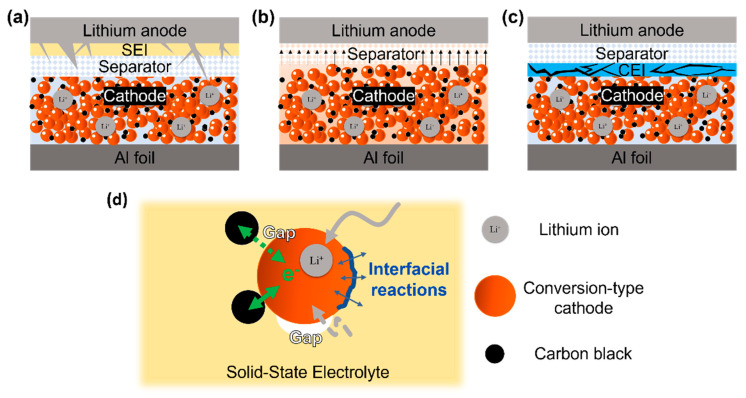
The failure mechanisms of conversion-type cathodes in a liquid electrolyte: (**a**) lithium dendrite formation on the anode, (**b**) cathode active material dissolution, and (**c**) fractured cathode electrolyte interface. (**d**) Schematic drawing of possible interfacial conditions in the cathode region of a solid-state lithium battery with a conversion-type cathode.

In a traditional lithium battery configuration with a conversion-type cathode and a liquid electrolyte, there are several scenarios that can lead to battery failure, as shown in Figure 7. On the anode side, during repeated cycling, dendritic lithium can form in the liquid electrolyte, potentially penetrating the separator and causing a short circuit (Figure 7a). On the cathode side, some conversion-type cathode materials can dissolve in the electrolyte during redox reactions, resulting in capacity loss and cathode structural instability (Figure 7b). The soluble active material species may further shuttle to the lithium metal anode, leading to rapid degradation. Brittle and fragile CEI could also form in conversion-type cathode systems, which would fracture and thicken further due to cathode swelling and shrinkage (Figure 7c). A thick and damaged CEI will significantly increase the impedance and thereby deactivate the cathode. The above challenges can be solved by introducing a solid electrolyte to replace the liquid electrolyte. Undoubtedly, lithium dendrite formation can be greatly suppressed, and the solid-state electrolyte can also act as a rigid separator to prevent short-circuiting. In addition, both the dissolution of the cathode active material and the formation of the CEI layer can be suppressed in the solid-state battery, so a stable cathode structure can be achieved.

While the solid-state electrolyte system appears to resolve several critical problems associated with the conversion-type cathodes in lithium batteries, there may be several concomitant problems that come with its implementation. First of all, in case the attachment between the carbon black and the cathode material surface is weak, the resulting electron contact resistance can be high (green arrow in Figure 7d). Second, the movement of lithium ions occurs through the channels established by the solid electrolyte, and the presence of any gaps between the electrolyte and cathode material can potentially render the cathode inactive or impair its efficiency (white gap in Figure 7d). Moreover, the transition metals present in chalcogenide and halide cathode materials can act as catalysts that degrade the solid-state electrolyte at the interface, resulting in unstable redox reactions (blue interface in Figure 7d). As a result, the challenges of interfacial instability, volume change, and chemical incompatibility should be effectively addressed through smart cell configuration and meticulous electrode preparation.

The development of commercial solid-state batteries is still a long way off, and solid-state batteries with conversion-type cathodes will require extensive efforts to become practical. However, solid-state Li–S cells have emerged as a promising advanced battery system due to the elimination of polysulfide dissolution and have attracted tremendous attention in recent years. Although there is not much work on solid-state battery design focusing on sulfide or fluoride cathodes, both of these cathode systems integrated into solid-state batteries have the potential to achieve high energy density if carefully designed. A rationally designed cell configuration can effectively improve the utilization of active materials, rate performance, and cycle life, which requires continuous research and development efforts in the future.

## Figures and Tables

**Table 1:** Summary of the solid-state battery configurations with various conversion-type cathodes.

Cell Configuration	Temp.	Capacity	Rate	Cycle Life	Feature	Ref.
**Chalcogen Cathode**						
rGO@S‖Li_10_GeP_2_S_12_‖75Li_2_S/24P_2_S_5_/1P_2_O_5_‖Li	60 °C	830 mAh g^−1^	1 C	750	The conformal S coating minimizing interface resistance & stress/strain	[66]
80Li_2_S/20LiI+LPS‖LPS+Kevlar‖Li	25 °C	537.8 mAh g^−1^	0.2 C	100	Thick cathode-supported all-solid-state lithium batteries	[67]
S@LLZO@C‖PEO-LiClO_4_‖Li	37 °C	>900 mAh g^–1^	N/A	90	A LLZO nanoparticle-decorated porous carbon foam for high S utilization	[68]
Li_2_S_6_‖LYZP‖Li	25 °C	≈1000 mAh g^−1^	0.2 C	150	A NaSICON solid-electrolyte/separator suppressing polysulfide crossover	[78]
Se+Li_3_PS_4_‖Li_3_PS_4_‖Li or LiSn alloy	25 °C	652 mAh g^−1^	50 mA g^−1^	100	The Se cathode improving charge transfer in solid-state batteries	[87]
Se‖Li_7_La_3_Zr_2_O_12_‖Li	465 °C	824 mAh g^−1^	30 mA g^−1^	N/A	A high-temperature molten Li-Se battery for stable OCV and cyclability	[88]
SeS_2_‖Li_10_GeP_2_S_12_+Li_3_PS_4_‖Li	25 °C	1100 mAh g^−1^	50 mA g^−1^	100	SeS_x_ solid solutions introduced into S cathode for enhanced utilization	[89]
LPSCB-MWCNTs‖Li_6_PS_5_C_l0.5_Br_0.5_‖Li-In	25 °C	12.56 mAh cm^−2^	≈0.7 C	1030	The electrochemically decomposed LPSCB forming a multiphase cathode	[90]
**Chalcogenide Cathode**						
FeS_2_‖99.5(70Li_2_S/30P_2_S_5_)/0.5FeS_2_‖Li–In	25 °C	543 mAh g^−1^	0.03 mA cm^−2^	20	A FeS_2_-doped solid electrolyte lowering interfacial resistance	[100]
Co_0.1_Fe_0.9_S_2_‖Li_10_GeP_2_S_12_/75Li_2_S/24P_2_S_5_/1P_2_O_5_‖Li	N/A	685.8 mAh g^−1^	500 mA g^–1^	100	The catalytic cobalt in FeS_2_ cathode enhancing electrochemical activity	[101]
S+VS_2_+Li_3_PS_4_‖Li_3_PS_4_‖Li-In	25 °C	7.8 mAh cm^−2^	0.12 mA cm^−2^	200	The hybrid S/VS_2_ cathode achieving high sulfur utilization	[108]
TiS_2_+75Li_2_S/25P_2_S_5_‖75Li_2_S/25P_2_S_5_‖Li_0.5_In	30 °C	837 mAh g^−1^	50 mA g^−1^	60	An amorphous Li-Ti-P-S phase offering increased capacity	[109]
**Halide Cathode**						
FeF_3_‖IL@SPF+LATP‖Li	25 °C	524.3 mAh g^−1^	0.1 C	100	A conformal sericin protein film stabilizing the Li-LATP interface	[128]

## Data Availability

All collected data are presented in the manuscript.
